# Supplementary material for: Covariance matrix filtering with bootstrapped hierarchies
Source: PLoS One. 2021 Jan 14;16(1):e0245092. doi: 10.1371/journal.pone.0245092 (PMC7808632; doi:10.1371/journal.pone.0245092)
Supplement: S1 Appendix — (PDF) [file pone.0245092.s001.pdf]

Supporting Information of Covariance matrix filtering with bootstrapped hierarchies

Christian Bongiorno, Damien Challet

January 5, 2021

Hierarchical Clustering Average Linkage-Filtered Correlation Matrix (HCAL)

The Notation

We describe in this section the strictly hierarchical method of Ref [1]. Given a generic matrix $R \in \mathbb{R}^{n \times t}$, a generic σ_{ij} element of the $n \times n$ sample covariance matrix is defined as

$$\sigma_{ij} = \frac{1}{t} \sum_{h=1}^{t} (r_{ih} - \bar{r}_i)(r_{jh} - \bar{r}_j) \tag{1}$$

where $\bar{r}_i = \sum_{h=1}^{t} r_{ih}/t$ is the sample mean. The related Pearson correlation coefficient is defined as

$$c_{ij} = \frac{\sigma_{ij}}{\sqrt{\sigma_{ii}\,\sigma_{jj}}} \tag{2}$$

Hierarchical Clustering

Hierarchical clustering is an agglomerative (bottom-up) algorithm that recursively clusters groups of objects according to a distance. The latter is defined in the simplest way: the elements Pearson correlation matrix C is transformed into distance matrix D defined as

$$d_{ij} = 1 - c_{ij}, \tag{3}$$

which respects the axioms of a distance. Then a distance metric among clusters must by defined: in the HCAL case, it is based on the average linkage between clusters p and q

$$\rho_{pq} = \frac{\sum_{i \in \mathfrak{C}_p} \sum_{j \in \mathfrak{C}_q} d_{ij}}{n_q\, n_p}, \tag{4}$$

where $\mathfrak{C}_p$ and $\mathfrak{C}_q$ are the sets of elements belonging to the clusters p and q respectively, and n_p and n_q are their cardinality.

Hierarchical clustering works as follows: initially, each element has its own cluster. Then, the pair of clusters (p, q) with the smallest distance (i.e., largest correlation) ρ_{pq} are merged to form a new cluster s such that $\mathfrak{C}_s = \mathfrak{C}_p \cup \mathfrak{C}_q$. The algorithm recursively joins a pair of clusters until all the nodes fall into a single unique cluster. The genealogy $\mathfrak{G}$ of the hierarchical clustering can be uniquely identified by the sequence of $n - 2$ joins among the pairs of clusters identified by the method, and this defines a dendrogram (also known as a tree).

Algorithm 1: Hierarchical Clustering Average Linkadge Filtering

Result: HCAL filtered matrix
$C^{<} \leftarrow \emptyset \in \mathbb{R}^{n\times n}$;
$D \leftarrow 1 - C$;
set($\mathfrak{C}$) $\leftarrow \{\{1\},\{2\},\cdots\{n\}\}$;
while $|set(\mathfrak{C})| > 1$ **do**
  Compute ρ_{pq} for all pair of clusters in set($\mathfrak{C}$);
  Find (p,q) with lowest ρ_{pq} ;
  $c^{<}_{ij} = c^{<}_{ji} \leftarrow 1 - \rho_{pq}\ \forall\, i \in \mathfrak{C}_p,\, j \in \mathfrak{C}_q$;
  $\mathfrak{C}_s \leftarrow \mathfrak{C}_p \cup \mathfrak{C}_q$;
  set($\mathfrak{C}$) $\leftarrow$ set($\mathfrak{C}$) $\cup \{\mathfrak{C}_s\}$;
  set($\mathfrak{C}$) $\leftarrow$ set($\mathfrak{C}$) $- \{\mathfrak{C}_p, \mathfrak{C}_q\}$;
end
$c^{<}_{ii} \leftarrow 1\ \forall\, i \in 1,2,\cdots n.$

Filtering correlation matrices with Hierarchical Clustering Average Linkage (HCAL)

Hierarchical clustering does not filter out noise from the correlation matrix, but merely yields a tree-like structure of dependence. Ref. [1] proposes to clean the correlation matrix from its hierarchical structure: whenever one merges two clusters, one replaces the correlation coefficients among the elements of these two clusters by their average value. Mathematically, for each sub-matrix defined from the indices $\mathfrak{F}_{pq} = \{(i,j) : i \in \mathfrak{C}_p,\, j \in \mathfrak{C}_q\}$, one replaces all its elements with their average: mathematically one builds a matrix $C^{<}$ with elements

$$c^{<}_{ij} = c^{<}_{ji} = 1 - \rho_{pq} \quad \text{where} \quad (p,q) \in \mathfrak{G},\ (i,j) \in \mathfrak{F}_{pq}, \tag{5}$$

ρ_{pq} is the average distance between clusters p and q (see (4)) and the diagonal of $C^{<}$ is set to 1, see Algorithm 1 for the meta-code. An equivalent description of this approach is in terms of the factor loading matrix, as in the original paper [1].

It is important to stress that the matrix $C^{<}$ will be positively defined by construction [1]. The main feature of this model is to obtain the simplest matrix $C^{<}$ that shares the same dendrogram as C; this means that by applying the HCAL to both C and $C^{<}$, the resulting dendrograms will be identical. However, we believe that this is also one of the main limitations of this approach as it does not account for the possible presence of overlap among clusters.

Bootstrap Average Linkage Correlation Matrix

To overcome these two issues of HCAL filtering while keeping its advantages, we propose here a new approach to filter correlation matrices based on data matrix bootstrap resampling of the feature indices; therefore, it better accounts for the influence of randomness on the inferred structure. We call it BAHC, which stands for Bootstrap-averaged hierarchical clustering.

Our recipe prescribes to create a set of m bootstrap copies of the data matrix R, denoted by $\{R^{(1)}, R^{(2)}, \cdots, R^{(m)}\}$. A single bootstrap copy of the data matrix $R^{(b)} \in \mathbb{R}^{n\times t}$ is defined entry-wise as $r^{(b)}_{ij} = r_{is^{(b)}_j}$, where $\mathbf{s}^{(b)}$ is a vector

of dimension t obtained with random sampling by replacement of the elements of the vector $\{1, 2, \cdots, t\}$. The vector $\mathbf{s}^{(b)}$, $b = 1, \cdots, m$ are independently sampled.

Each bootstrap copy b of the data matrix has an associated Pearson correlation matrix $C^{(b)}$ from which we can construct the HCAL filtered matrix $C^{(m)<}$. Finally, each element of the filtered Pearson correlation matrix C^{BAHC} is defined as the average over the m filtered bootstrap copies, i.e.,

$$c_{ij}^{\mathrm{BAHC}} = \sum_{b=1}^{m} \frac{c_{ij}^{(b)<}}{m} \tag{6}$$

We stress that when $c_{ij}^{(h)} > 0$ for all (i, j) then $C^{(h)<}$ are positive-definite matrices. As result C^{BAHC} is also a positive defined matrix. Note that we never obtained a non positive-definite $C^{(h)<}$ in all our tests, even when some correlation coefficients are negative; we have not been able to find a mathematical proof in this case.

The main advantage of the BAHC method is not to force C^{BAHC} to be embedded in a purely hierarchical structure. Indeed, different bootstraps may yield different dendrograms, in which case a strict hierarchical structure is too stringent. Thus, the BAHC method can reproduce some degree of overlap among clusters defined in a hierarchical way.

Eigenvector in- and out-of-sample overlap from the Oracle estimator

We recall the concept of Oracle estimator Ξ: given the spectral decomposition of the in-sample correlation matrix $C^{in} = U^{in}\Lambda^{in}U^{in\dagger}$ and the spectral decomposition of the out-of-sample correlation matrix $C^{out} = U^{out}\Lambda^{out}U^{out\dagger}$, where $\Lambda^{in/out}$ are diagonal eigenvalue matrices made from the eigenvalues of $C^{in/out}$, and $U^{in/out}$ is the matrix defined by the eigenvectors of $C^{in/out}$, the Oracle eigenvalue matrix is defined as

$$Z^{in} = \left(U^{in\dagger}C^{out}U^{in}\right)_d \tag{7}$$

where the superscript in indicates that we used the in-sample eigenvectors for its estimation. The operator $()_d$ sets to zero all the off-diagonal elements. Then the Oracle estimator of the correlation matrix is defined as

$$\Xi^{in} = U^{in}Z^{in}U^{in\dagger}. \tag{8}$$

Ref. [2] shows that Oracle eigenvalues are the optimal correction of the in-sample eigenvalues Λ^{in} in the sense that it minimizes the Frobenius norm of the difference between the out-of-sample correlation matrix and the corrected in-sample one $\left\|C^{out} - \Xi^{in}\right\|_F$. Although this estimator sounds worryingly tautological, since it require the knowledge of the out-of-sample correlation to construct the most similar estimator, Ref. [2] shows that is possible to obtain Z^{in} in the $t, n \to \infty$ at constant $q = n/t$ limit without the knowledge of C^{out} for a broad set of distributions and noises (multiplicative and additive) if the system is stationary and for $t > n$ (low-dimensional regime). Indeed, it easy to show that

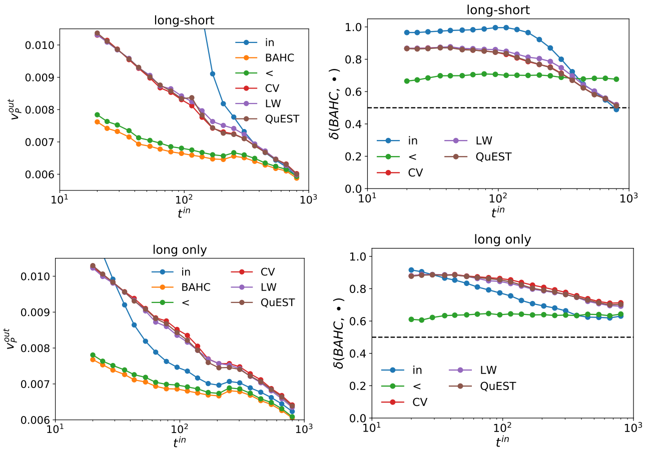

Figure 1: Left plots: realized risk for different estimators; right plots: fraction of time the realized risk of BAHC is smaller than the one obtained with alternative estimators. 10,000 independent simulations per point; $t^{out} = 42$ days, $n = 100$ assets, Hong Kong equities.

the Oracle estimator is exactly C^{out} if and only if $U^{in} = U^{out}$ since

$$Z^{in} = \left(U^{in\dagger} C^{out} U^{in}\right)_d = \left(U^{in\dagger} U^{out} \Lambda^{out} U^{out\dagger} U^{in}\right)_d = \\ = \left(U^{out\dagger} U^{out} \Lambda^{out} U^{out\dagger} U^{out}\right)_d = \left(\Lambda^{out}\right)_d = \Lambda^{out}. \quad (9)$$

Therefore the Frobenius norm of $\left\|\Xi^{in} - C^{out}\right\|_F$ can be interpreted as a measure of the overlap between the out-of-sample eigenvectors U^{out} and the in-sample ones U^{in}. See [3] for an alternative measure of eigenvector overlap.

Global minimum-variance portfolios in other equity markets

Figures 1 and 2 report the out-of-sample risk of covariance matrix cleaning methods with the same set up for Hong Kong stock exchange 1. The analysis cover 1281 stocks in from 2005-10-19 to 2017-06-23. The stocks are not simultaneously listed over all time-period: the number stocks ranges from 590 on 2008-08-22 to 1277 on 2017-06-14. Results are qualitatively consistent with those observed in the US equity market. List of tickers and scripts to download data available on request.

Global minimum-variance portfolios with out-of-sample standard deviation

In order to isolate the effect of the correlation matrix filtering we computed the portfolio composition by considering the out-of-sample standard deviation of the single equities. However, we did not observe any qualitative difference.

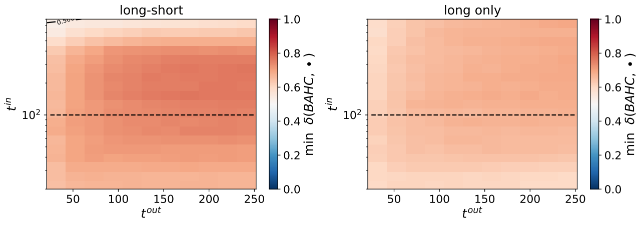

Figure 2: Fraction of time the realized risk of BAHC is smaller than the best performing alternative method. Left plot: portfolios with long and short positions, right plot: portfolios with only long positions. The level curve corresponds to a 50% probability. 10,000 independent simulations per point; (a) to (d) : $t^{out} = 42$ days, $n = 100$ assets

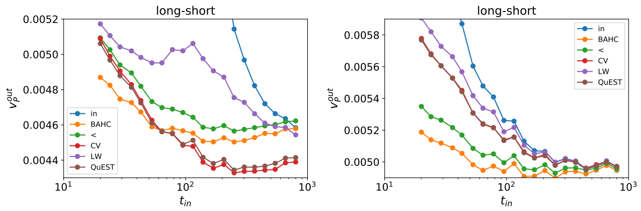

Figure 3: Realized risk on Russell1000 of different covariance estimator. The out-of-sample standard deviations of single equities was used here to compute the portfolio compositions. Left plot: portfolios with long and short positions, right plot: portfolios with only long positions. 10,000 independent simulations per point; $t^{out} = 42$ days, $n = 100$ assets

References

[1] Tumminello M, Lillo F, Mantegna RN. 2007 Hierarchically nested factor model from multivariate data. *EPL (Europhysics Letters)* **78**, 30006.

[2] Bun J, Allez R, Bouchaud JP, Potters M. 2016 Rotational invariant estimator for general noisy matrices. *IEEE Transactions on Information Theory* **62**, 7475–7490.

[3] Bun J, Bouchaud JP, Potters M. 2018 Overlaps between eigenvectors of correlated random matrices. *Physical Review E* **98**, 052145.
